# Revealing the diagnostic value and immune infiltration of senescence-related genes in endometriosis: a combined single-cell and machine learning analysis

**DOI:** 10.3389/fphar.2023.1259467

**Published:** 2023-10-03

**Authors:** Lian Zou, Lou Meng, Yan Xu, Kana Wang, Jiawen Zhang

**Affiliations:** ^1^ Chongqing Emergency Medical Center, Department of Obstetrics and Gynecology in Chongging University Central Hospital, Chongqing, China; ^2^ Department of Gynecology, West China Second Hospital of Sichuan University, Chengdu, China

**Keywords:** machine learning, immune infiltration, endometriosis, senescence-related genes, aging, integrative bioinformatics, senescence-associated molecular

## Abstract

**Introduction:** Endometriosis is a prevalent and recurrent medical condition associated with symptoms such as pelvic discomfort, dysmenorrhea, and reproductive challenges. Furthermore, it has the potential to progress into a malignant state, significantly impacting the quality of life for affected individuals. Despite its significance, there is currently a lack of precise and non-invasive diagnostic techniques for this condition.

**Methods:** In this study, we leveraged microarray datasets and employed a multifaceted approach. We conducted differential gene analysis, implemented weighted gene co-expression network analysis (WGCNA), and utilized machine learning algorithms, including random forest, support vector machine, and LASSO analysis, to comprehensively explore senescence-related genes (SRGs) associated with endometriosis.

**Discussion:** Our comprehensive analysis, which also encompassed profiling of immune cell infiltration and single-cell analysis, highlights the therapeutic potential of this gene assemblage as promising targets for alleviating endometriosis. Furthermore, the integration of these biomarkers into diagnostic protocols promises to enhance diagnostic precision, offering a more effective diagnostic journey for future endometriosis patients in clinical settings.

**Results:** Our meticulous investigation led to the identification of a cluster of genes, namely BAK1, LMNA, and FLT1, which emerged as potential discerning biomarkers for endometriosis. These biomarkers were subsequently utilized to construct an artificial neural network classifier model and were graphically represented in the form of a Nomogram.

## 1 Introduction

Endometriosis (EM) is one of the most prevalent chronic inflammatory gynecological maladies, affecting nearly 10% of women in the stage of childbearing maturation. Its effects extend to the quality of life and overall wellbeing of a significant number of women (Liang and Yao, 2016; Becker et al., 2022). Its principal indications are pelvic discomfort, dysmenorrhea, and uterine hemorrhaging. Such symptoms affect nearly half of patients, leading to infertility and other complications (Eskenazi and Warner, 1997; Giudice and Kao, 2004; Meuleman et al., 2009). EM has emerged as a public health concern, putting substantial pressure upon women and their families. Nonetheless, there are insufficient precise diagnostic and therapeutic interventions. The pelvic discomfort characteristic of EM can be erroneously identified as abdominal pain originating from the menstrual cycle, thereby confounding the diagnostic process. Moreover, there is a consistent period of latency spanning 7–11 years, from the onset of symptomatic presentation to the eventual moment of definitive diagnosis (Greene et al., 2009; Rogers et al., 2013). Laparoscopic surgery is a benchmark for the diagnosis of EM. However, it is not widely supported as a diagnostic modality due to its considerable financial burden and potentially adverse effects on the physiological state of women (Giudice and Kao, 2004; Li et al., 2021).

A complete understanding of the pathogenesis underlying EM remains elusive. Several factors, including retrograde menstruation, cytokine involvement, estrogen influence, inflammatory reactions, and ectopic implantation, are considered potential etiological contributors. Among these, the process of epithelial–mesenchymal transition (EMT) is gaining recognition in research (Zondervan et al., 2018). Although the mechanism of the senescence genes in EM is not fully comprehended, endometrial epithelial cells are often characterized by senescence in lesions (Velarde and Menon, 2016; Brighton et al., 2017). By applying bioinformatic analyses, such as enrichment evaluations, a significant nexus between EM and the cellular cycle has been established. Notably, the affirmative enrichment of the cellular cycle within the context of EMT concurs with the cycle being intrinsically linked to genes associated with senescence. Furthermore, it has been found that the expression of SRGs is attenuated in the context of EM (Wang et al., 2022). Telomere correlation analysis also revealed that the average telomere length is shorter in women with EM (Gleason et al., 2022). Cellular senescence (CS) is a stress-induced response to various biological signals that results in stable cell-cycle arrest (Salama et al., 2014). CS is often associated with DNA damage, and DNA damage kinase is associated with telomere shortening (d'Adda di Fagagna, 2008; Harley et al., 1990).

Given such interrelations, SRGs show considerable promise as candidates for EM diagnosis. This investigation made a strategic choice to amalgamate the dataset of SRGs with the EM dataset obtained from both single-cell sequencing repositories and diverse transcriptomic compilations culled from public databases. Such integration facilitated comprehensive bioinformatic analyses, the formulation of diagnostic models, and the execution of single-cell scrutiny. We thereby corroborate the diagnostic potential of SRGs within the context of EM.

## 2 Materials and methods

### 2.1 Collection of gene microarray data and data pre-processing for endometriosis

The Gene Expression Omnibus (GEO) database, hosted on NCBI (https://www.ncbi.nlm.nih.gov/geo/), supplied three gene expression microarray datasets related to EM: GSE12768-GPL7304 (Borghese et al., 2008), GSE11691-GPL96 (Hull et al., 2008), and GSE7305-GPL570 (Hever et al., 2007). For comprehensive details concerning these datasets, see Supplementary Table S1. We included a cohort of patients who had been definitively diagnosed with EM from the GEO database and transcribed and analyzed their normal and diseased endometrial human tissue. In addition, cohorts with complete patient follow-up information and complete clinical information, and cohorts with complete micro-matrix data, were required to ensure data quality for subsequent bioinformatics analysis. To minimize the influence of other gynecological conditions, we excluded subjects who may have concurrently suffered from other important gynecologic conditions to maintain the cohort's integrity. To facilitate agreement with official platform annotations, probe IDs were transformed into gene symbols, wherein probes with multiple associated gene symbols and those without were systematically excluded. For instance, when multiple gene symbols were ascribed to a single gene, the median value was computed to represent the unique expression value. Datasets GSE12768 and GSE11691 were thus merged, and, to mitigate the influence of batch disparities within the microarray expression data, the ComBat function encompassed within the R package “sva” was used for batch effect rectification (Roder et al., 2019). The resulting processed microarrays were subsequently employed as the intrinsic training dataset, while GSE7305 functioned as the extraneous validation dataset. For this research, 307 SRGs (SRGs) were extracted from the Human Senescence Genome Resource (HAGR, http://genomics.senescence.info/genes/) (Tacutu et al., 2018). The complete enumeration of these genes is provided in Supplementary Table S2.

### 2.2 Identification of differentially expressed SRGs

The gene expression microarrays specific to EM were intersected with the set of 307 SRGs, revealing their shared genes. This common gene pool was subsequently subjected to differential analysis. To facilitate this, the “limma” R package was employed, adopting established thresholds (*p*-value <0.05 and Log_2_|FC| > 0.5) to discriminate genes displaying differential expression (Song et al., 2022a; Zhao et al., 2022; Song et al., 2022b). A selection of 45 genes, termed differentially expressed SRGs (DE-SRGs), was thus culled from this analysis.

The “ggplot2” and “pheatmap” R packages were used to present these outcomes (Chi et al., 2022a; Shen et al., 2022; Huang et al., 2023). The former created a visually informative volcano plot, while the latter constructed a heatmap depicting the expression patterns of the DE-SRGs. Additionally, deviation maps were formulated to underscore the divergences in gene expression tendencies between the EM and healthy cohorts. This was achieved using the “ggpubr” R package (version 4.0.2) (https://github.com/kassambara/ggpubr). These deviation maps delineate the trajectory of gene expression profiles within both the EM and healthy groups.

### 2.3 Functional enrichment analysis of differentially expressed SRGs

Utilizing the clusterProfiler R package (Jin et al., 2022; Chi et al., 2023a; Ren et al., 2023) (https://bioconductor.org/packages/release/bioc/html/clusterProfiler.html), a comprehensive enrichment analysis was executed for the DE-SRGs in the context of EM. This analysis interrogated the Kyoto Encyclopedia of Genes and Genomes (KEGG) pathways, Gene Ontology (GO) terms, and Disease Ontology (DO). The objective was to identify statistically significant biological functionalities and enrichment pathways, with strict criteria set at *p*-values <0.05 and Q-values <0.05 to ensure robustness. Prior to enrichment analysis, preprocessing was undertaken to ensure uniform gene annotation by transforming all gene symbols into Entrez IDs, which was facilitated by the human genome-wide annotation R package “org.Hs.e.g.,.db.” GO comprises three distinct components: biological process (BP), cellular component (CC), and molecular function (MF).

### 2.4 Feature gene identification by integrating three machine learning algorithms

The cohort of 45 DE-SRGs underwent a meticulous screening process whereing three distinct machine learning algorithms were harmoniously amalgamated: support vector machine-recursive feature elimination (SVM-RFE), random forest (RF), and the least absolute shrinkage and selection operator (LASSO) (Chi et al., 2023b; Song et al., 2023; Zhang et al., 2023). SVM-RFE, an advance upon the sequential backward selection algorithm rooted in the SVM’s tenet of maximal margin, delivers superior and more proficient classification performance, particularly for high-dimensional datasets. The realization of SVM-RFE was facilitated using the “e1071” R package, a tool that identifies relevant attributes while discarding redundant ones (Mundra and Rajapakse, 2010). The RF algorithm embodies a high-precision and sensitivity-oriented integration technique that integrates multiple decision trees and amalgamates the outcomes of diverse classifiers into a unified decision, achieved through the utilization of the “randomForest” R package (Strobl et al., 2007). This investigation prioritized the relative significance among the 45(DE-SRGs, ultimately selecting candidate genes whose relative importance surpassed the threshold of 0.1. Due to its reputation for proficient dimensionality reduction, LASSO emerged as an exceptional tool. To optimize its performance, a rigorous assessment was undertaken involving ten-fold cross-validations, orchestrated through the “glmnet” R package, to discern the optimal tuning parameter (*λ*) (Friedman et al., 2008). The optimal configuration of *λ* was discerned as the point of minimal magnitude. In comparison with alternative algorithms, LASSO was very efficient at evaluating high-dimensional datasets. The common genes extracted through the amalgamation of these three machine-learning algorithms were subsequently harnessed as biomarkers for the prediction of endometriosis.

### 2.5 Building an artificial neural network disease classification model and nomogram

Artificial neural networks (ANNs) are important in the field of deep learning within artificial intelligence systems, serving as consequential analytical instruments. Typically comprising three strata, ANN models encompass an input layer for information reception, one or more hidden layers for information processing, and an output layer for result computation. In this investigation, the “neuralnet” (Beck, 2018) R package was employed to construct ANN models founded upon three SRGs. These were orchestrated with a configuration comprising five hidden layers, and the gene weight data were harnessed to formulate a classification model primed for EM disease prediction. Similar parameters and processes were extended to an external dataset for model validation. ROC curves were computed employing the “pROC” R package to elicit AUC values, which are pivotal in evaluating the predictive efficacy of the ANN model. In parallel, a nomogram based on the three SRGs was meticulously formulated through the “rms” R package. Calibration curves and decision curve analysis (DCA) were used to visualize the discriminatory capacity of the model.

### 2.6 Network construction and functional enrichment analysis of WGCNA co-expression

The WGCNA algorithm clusters highly correlated genes into gene set modules, guided by a scale-free topological criterion and reveals the intricate interplay between these modules and disease traits. For this research, the “WGCNA” R package (Langfelder and Horvath, 2008) was pivotal, independently making connections between phenotypes (EM or health) and individual modules. The module exhibiting the highest correlation coefficient coupled with the most statistically significant *p*-value emerged as the principal module associated with endometriosis.

Subsequently, the genes nested within the pivotal modules were subjected to enrichment analysis which utilized Metascape data (http://metascape.org) (Zhou et al., 2019). The outcomes of this analysis highlighted the top 100 results from the enrichment, and a network diagram was generated that employed the top 20 clustering outcomes. In this diagram, the thickness of connecting lines served as an indicator of the similarity score, thus visually representing the intricate relationships established.

### 2.7 Immune cell infiltration abundance analysis

The R package IOBR (Zeng et al., 2021) (Immuno-Oncology Biology Research) amalgamated the four algorithms, TIMER, ESTIMATE, xCell, and CIBERSORT, which served as pivotal tools for quantifying the presence of immune cell infiltrations. In the context of this inquiry, the CIBERSORT algorithm emerged as the preferred choice, delineating the relative composition of 22 immune cell types. This characterization was achieved by discerning gene expression patterns in samples, ensuring that the cumulative presence of each immune cell type within a singular sample equated to 1. Spearman correlation was harnessed to unravel the intricate associations, enabling an exploration of the linkages between the three SRGs and the various constituents of the immune system.

### 2.8 Data processing and analysis of scRNA-seq

The 10× scRNA-seq data were processed as follows: 1) 10× scRNA-seq data were converted to Seurat objects using the R software Seurat package (Macosko et al., 2015). 2) Counts were quality-controlled (quality control (QC)) by excluding low-quality cells based on mitochondrial or ribosomal gene percentages. 3) Screening of the top 2000 high variable genes after QC using the “FindVariableFeatures” function. 4) Principal component analysis (PCA) and uniform manifold approximation and projection (UMAP) were based on the 2000 genes for downscaling and cluster identification (Becht et al., 2018). 5) The “FindAllMarkers” function identified significant marker genes in different clusters by setting log_2_FC to 0.3 and minimum pct to 0.25. 6) Our analysis of cluster annotations was performed using the SingleR package in the R software (Aran et al., 2019). Next, we performed Fisher precision tests to identify potentially important cell types. We calculated FC values for each cell type in tumor and normal samples and identified cell types with FC > 4 or FC < 0.25, with *p* < 0.05 being the critical cell type.

## 3 Results

### 3.1 GEO expression data pre-processing and analysis of variance

**FIGURE 1:**
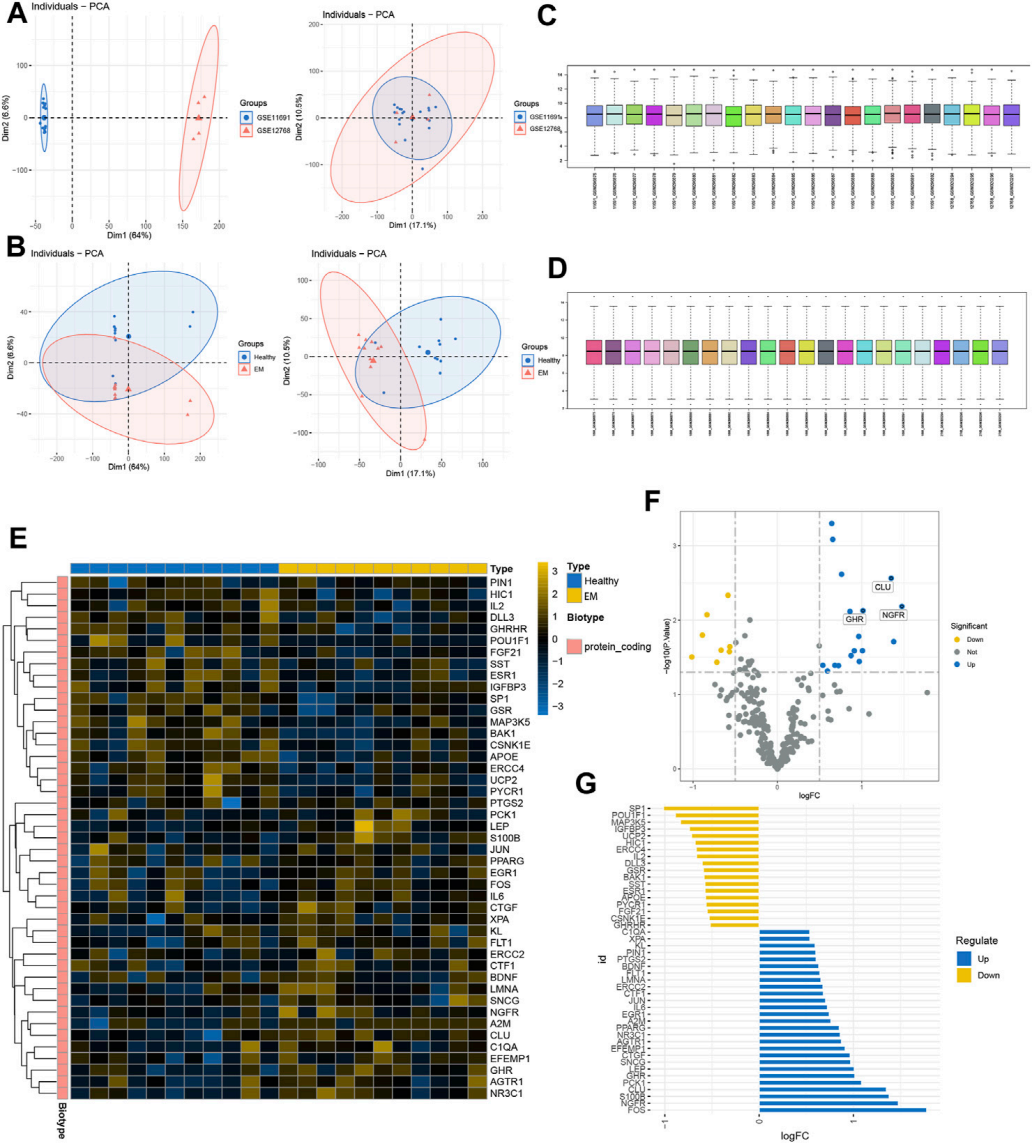
GEO expression data pre-processing. **(A, B)** Principal component analysis of EM versus control samples. **(C, D)** Box plots of raw data normalized between samples. **(E)** DEGs heat map plot. **(F)** DEGs volcano map plot. **(E)** DEGs butterfly plot. **(G)** DEGs deviation plot.

We first normalized the gene expression data of GSE11691 and GSE12768 with quantile normalization and merged the datasets with the R package “sva” to remove batch effects. Figure 1A shows a PCA of the two datasets before and after processing; as shown in the figure, the two datasets were initially separated without any intersection; after processing, their intersection can be used as a batch for subsequent analysis. Figure 1B shows the PCA results for the healthy and EM groups in the dataset, and Figures 1C, D show box plots of the two datasets after normalization—the different colors represent different samples and the columns indicate the gene expression values in the samples. We then performed disease differential gene analysis on the combined datasets and took the intersection with the collected senescence genes.

As shown in the figure, the intersection of the two datasets can be used as a batch of data for subsequent analysis. A total of 45 genes were identified as DEGs with 18 upregulated and 27 downregulated genes under the criteria of *p*-adjustment < 0.05 and log_2_ | fold-change (FC) | >0.5. Figure 1F shows the volcano map of the DEGs and the heatmap of the top 50 genes (Figure 1E). Finally, significantly different DEGs are displayed in the deviation plot (Figure 1G).

### 3.2 GO, KEGG, and DO enrichment analyses of senescence genes

**FIGURE 2:**
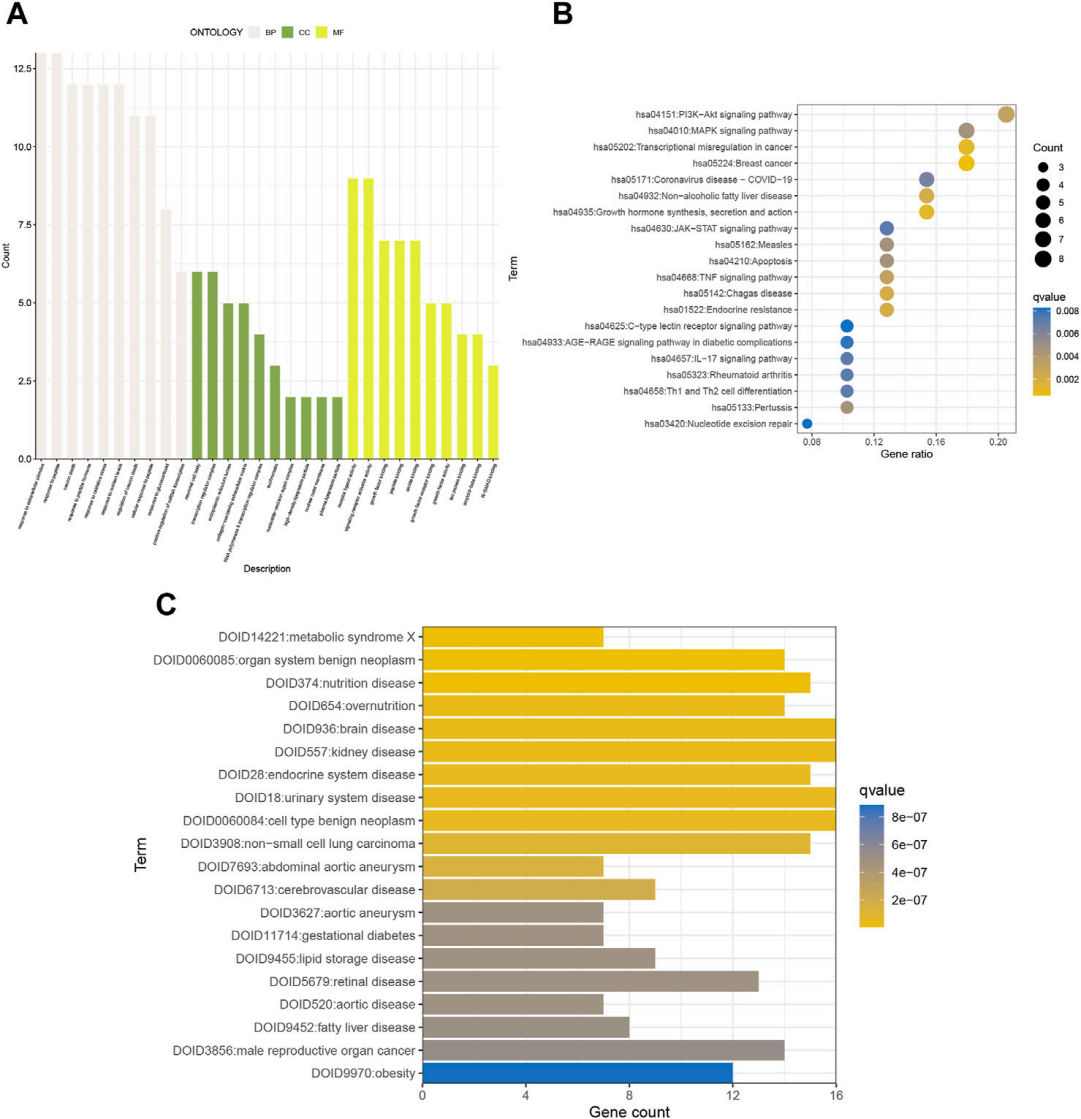
GO, KEGG, and DO enrichment analyses of senescence genes. **(A)** GO, **(B)** KEGG, and **(C)** DO analyses show the enriched biological functions of differentially expressed gene profiles.

We performed KEGG pathway analysis, GO functional analysis, and DO disease analysis to further evaluate the biological function of the differential genes. Consequently, we used *p*-value<0.01 and Q-value<0.05 as thresholds to identify items that were significantly enriched. Biological process (BP) includes response to extracellular stimulus, response to peptides, and the like. Cellular component (CC) includes neuronal cell body and transcriptional regulatory complex. Molecular function (MF) includes receptor ligand activity and signaling receptor activator activity (Figure 2A). KEGG analysis revealed enrichment in breast cancer, growth hormone synthesis, secretion, and action, transcriptional misregulation in cancer, and other pathways associated with gynecological cancers (Figure 2B). DO analysis found an endocrine system disease—urinary system disease (Figure 2C). In conclusion, the differential genes have a great impact on cellular receptive signaling-related functions and pathways and presumably may play a major role in disease mechanisms.

### 3.3 Cluster fusion of differential genes combined with clinical features

**FIGURE 3:**
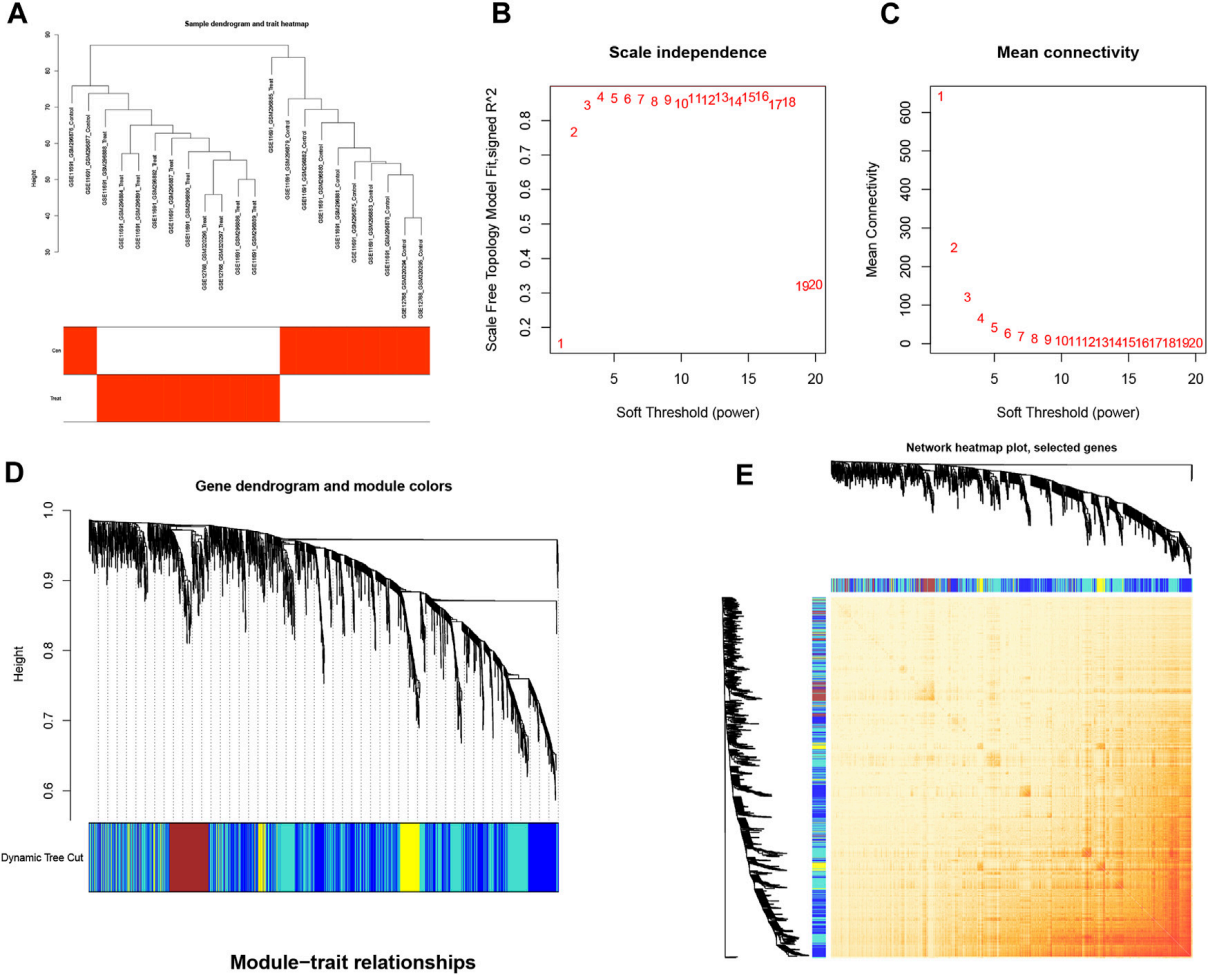
Cluster fusion of differential genes combined with clinical features. **(A)** Cluster dendrogram. **(B,C)** Analysis of network topologies for various soft-threshold powers. **(D)** Clustering dendrogram of DEGs with dissimilarity based on the topological overlap, together with assigned module colors. **(E)** Heatmap view of co-expressed genes in different modules in the top 1,500 genes.

We used WGCNA to analyze associations with clinical features of EM and differential genes, where the samples were clustered according to clinical features of the disease; the cohort was processed using the correlation coefficient method to obtain a sample clustering tree (Figure 3A). The samples were then analyzed by hierarchical clustering to construct a gene co-expression network with a soft threshold of 18 (R^2^ = 0.8) and higher average connectivity (Figures 3B, C). By clustering and fusing the characteristic genes into the differential genes and merging the highly similar modules, five modules were ultimately obtained (Figure 3D). By showing the relative independence of gene expression within these modules, the analysis of the combined results did not reveal any significant differences between the different modules (Figure 3E).

### 3.4 Identifying high-relevance modules and analyzing related functions

**FIGURE 4:**
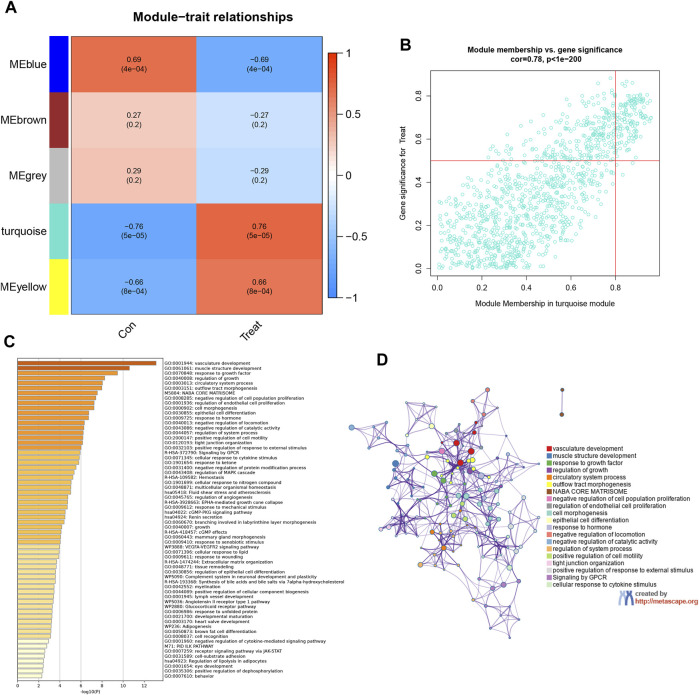
Identifying high-relevance modules and analyzing related functions. **(A)** Module–trait heatmap. Each row corresponds to a module eigengene; each column corresponds to a trait; each cell contains the corresponding correlation and *p*-value. **(B)** Scatterplot of gene significance. (GS) for weight vs. module membership (MM) in the brown module. There is a highly significant correlation between GS and MM in this module. **(C)** Heatmap of Metascape analysis colored by *p*-values. **(D)** Network of enriched sets colored by ID. Threshold: 0.3 kappa score; similarity score>0.3.

In an endeavor to quantify the interrelation between each module and the two clinical attributes, heatmaps were created to illustrate the intricate network of interconnections within the module features (Figure 4A). These heatmaps display the correlations between trait genes and the traits associated with obesity. Within this context, an exhaustive co-expression analysis of DEGs was undertaken, which identified the turquoise module as the epicenter of particularly robust correlation. This assertion was reinforced by scatter plots which yielded 106 hub genes that displayed an exceptionally high level of connectivity within the turquoise module (cor = 0.78, *p* < 1e-200) (Figure 4B). KEGG and GO enrichment analyses determined the functional significance of these 106 hub genes by employing the Metascape library. This identified over 100 prime genes with profound enrichment in lipid-related pathways and functions. These genes also demonstrated close associations with blood vessel establishment and morphological structures (Figures 4C, D). Such interplay strongly suggests that the hub genes may have a substantial impact on EM within hormone synthesis and action.

### 3.5 Three machine learning algorithms to screen for modeled genes

**FIGURE 5:**
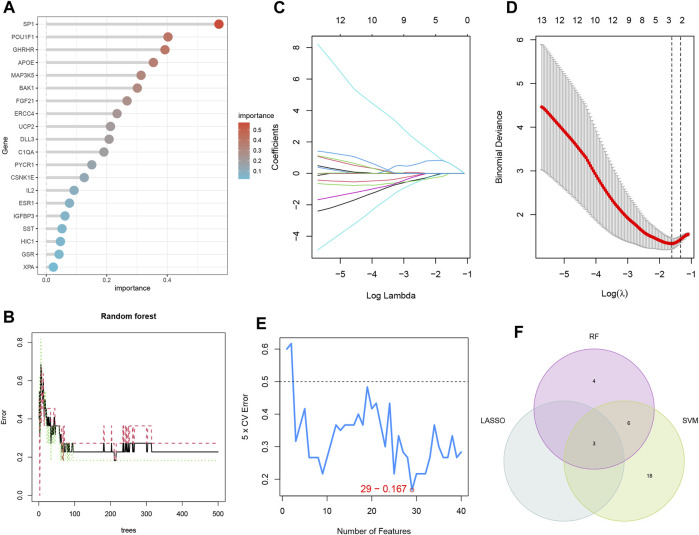
Three machine learning algorithms screen modeling genes. **(A)** Ranking of genes according to their relative importance. **(B)** Ten-fold cross-validation of tuning parameter selection in the LASSO model. Each curve corresponds to one gene. **(C)** LASSO coefficient profiles. The solid vertical line indicates the partial likelihood deviation SE. The dashed line is drawn at the best λ. **(D)** Random forest (RF) for the relationship between the number of trees and error rate. **(E)** Biomarker signature gene expression validation by support vector machine-recursive feature elimination (SVM-RFE). **(F)** LASSO, RF, and SVM-RFE algorithms shared by the Venn diagram of signature genes.

The LASSO, RF, and SVM-RFE algorithms were used to screen feature genes among the differentially expressed genes associated with key EM progression and senescence processes. For the LASSO algorithm, we selected after cross-validation the minimum criteria for constructing the LASSO classifier to identify three feature genes (Figures 5C, D); the error was minimized when the number of features was 29 (Figure 5E). Thus, 29 relevant feature genes were obtained, and the top 13 with importance greater than 0.1 were selected from the classification tree results (Figures 5A, B) in combination with RF feature selection. Through crossover, the three feature genes shared by LASSO, RF, and SVM-RFE algorithms were finally identified, namely, BAK1, FLT1, and LMNA, and their relationships were represented by the Venn diagram (Figure 5F).

### 3.6 Development and validation of ANN models and nomogram based on three SRGs

**FIGURE 6:**
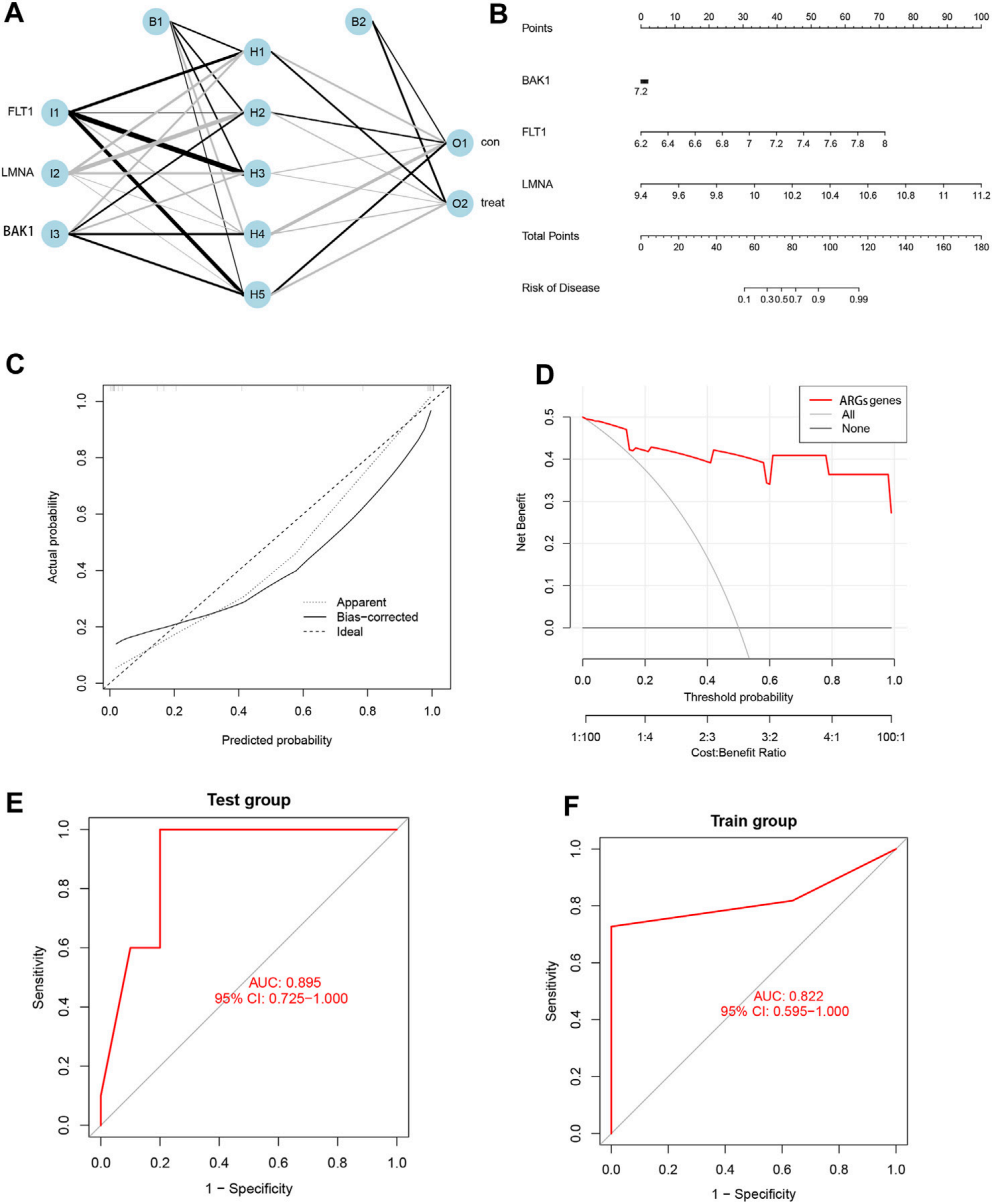
Development and validation of ANN models and nomogram based on three SRGs. **(A)** Artificial neural network plot. **(B)** Nomogram plot. **(C)** Calibration curve. **(D)** DCA curve. **(E)** Test group ROC curve. **(F)** Training group ROC curve.

We ultimately selected three pivotal SRGs to construct a hidden layer number 5 for the ANN model for clinical EM diagnosis (Figure 6A). The satisfactory reliability and accuracy of the prediction model were validated by the ROC curves, and it was found that the AUC values were greater than 0.85 in both the training (AUC = 0.822) and the validation cohorts (AUC = 0.895) (Figures 6E, F). To further promote the clinical application of the three SRG biomarkers in this study, nomogram plots were also drawn (Figure 6B) to calculate patient scores based on the expression data of the three biomarkers to infer the probability of prevalence in EM patients. In addition, both the DCA and calibration curves indicated a higher net benefit and accuracy of using the nomogram for diagnostic prediction in EM patients (Figures 6C, D).

### 3.7 Detection of immunological features in EM and normal samples

**FIGURE 7:**
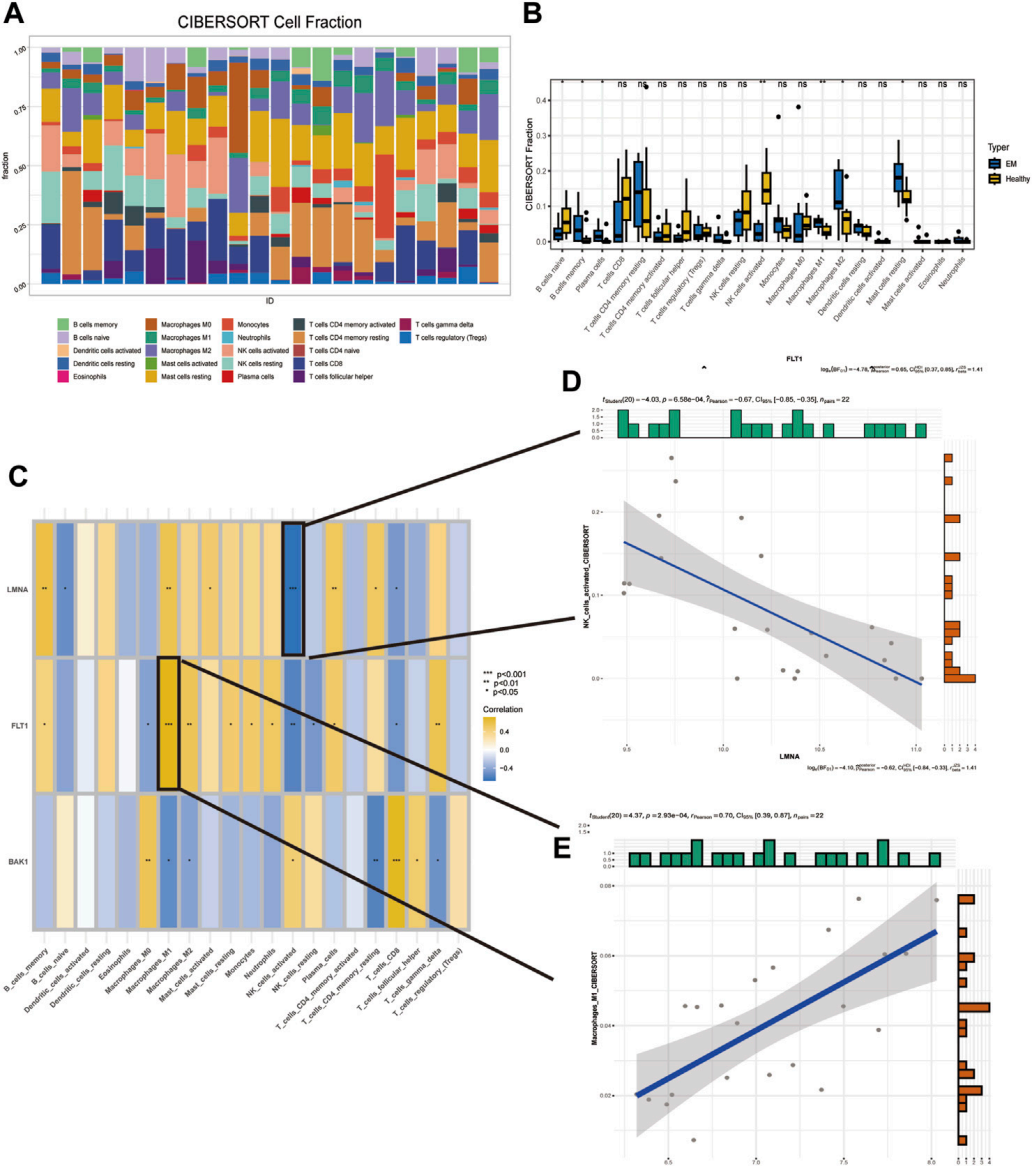
Detection of immunological features in EM and normal samples. **(A)** Immune infiltration stack plot. **(B)** Comparison of immune cells between EM patients and controls. **(C)** Correlation of three SRGs with immune cells. **(D)** Correlation of FLT1 with Macrophages_M1. **(E)** Correlation of LMNA with NK_cells_activated.

Immune cells play an essential role in endometrial shedding, tissue repair, and preventing infection (Shen et al., 2021), although a disproportionate number of immune cells in an abnormal immune environment is thought to play a key role in EM pathogenesis (Wu et al., 2017). The CIBERSORT algorithm in IOBR was used to calculate the proportion of 22 immune cell infiltrates in EM tissues. A stacked plot was used to fully visualize the distribution of the immune infiltrate in the EM tissue (Figure 7A), with the sum of all immune cell proportions being 1. A significant increase in B cells naive and NK cells activated in the patient’s uterine tissue was observed, while the proportion of immune cells such as macrophages m1 and m2 decreased significantly (Figure 7B). In addition, the correlation between the three SRGs and immune cells was explored based on the permanent method (Figure 7C), where FLT1 and Macrophages_M1 had the highest positive correlation while LMNA and NK_cells_activated had the highest negative correlation; their correlation scatter plots are shown in Figures 7D, E.

### 3.8 Single-cell analysis

**FIGURE 8:**
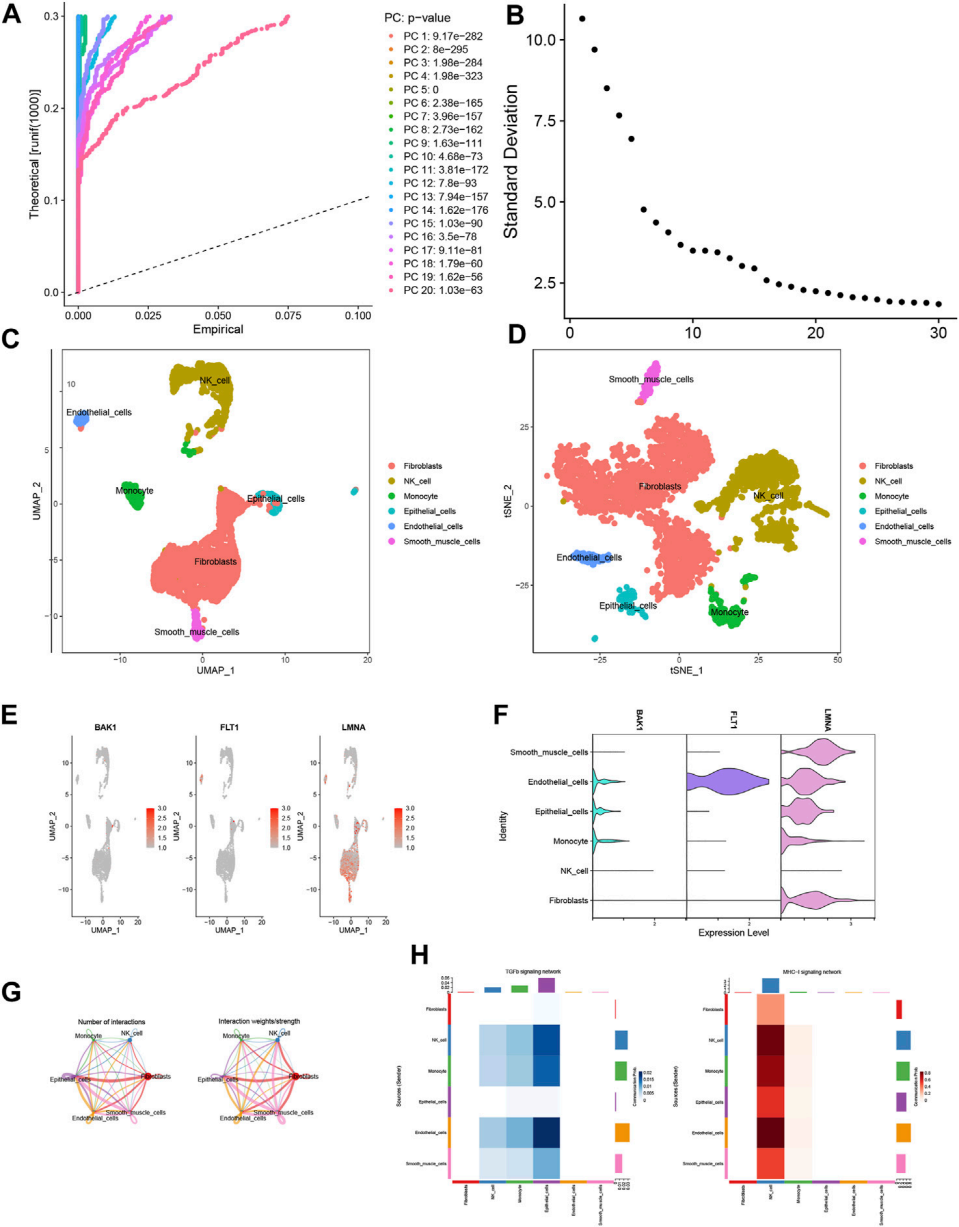
Single-cell analysis. **(A)** JackStrawPlot. **(B)** ElbowPlot. **(C,D)** Distribution of results after annotation of immune cell subpopulations. **(E,F)** Expression of modeled genes in each cell type. **(G)** Cellular communication network map. **(H)** Heatmap of the correlation between ligand receptors in TGFb and MHC-1 in various cell types.

Following quality control procedures applied to the single-cell dataset, a crucial phase was PCA-based dimensionality reduction, where the determination of the optimal number of selected PCs was substantiated by insights gleaned from the JackStrawPlot and ElbowPlot plot analyses (Figures 8A, B). Subsequently, the “FindClusters” function harnessed from the “Seurat” package was deployed to cluster the individual cells, which were further annotated using SingleR. The resulting visualization was realized through t-SNE and UMAP downscaling techniques, thereby culminating in the identification and characterization of six distinct cell types (Figures 8C, D). The exploration extended to evaluating the expression occupancy of the three modeled genes—BAK1, FLT1, and LMNA—within these six cell types. Intriguingly, the results revealed that LMNA exhibited heightened expression across nearly all cell types, whereas FLT1 demonstrated pronounced expression solely within epithelial cells (Figures 8E, F). We then explored deeper into ligand–receptor networks and the delineation of specific pathways, thus facilitating the inference of cell–cell communication networks. Based on these findings, MHC-I and TGFb emerged as prominent drivers of signaling communication within NK cells and epithelial cells, respectively (Figures 8G, H).

## 4 Discussion

Despite affecting approximately 10% of women within the reproductive age bracket, the intricate connection between EM and various health ramifications, including early natural menopause (ENM), remains enigmatic. A substantial corpus of clinically documented cases underscores the notion that EM is intrinsically related to ovarian senescence, thereby exerting a tangible influence on the temporal span leading to menopause (Pokoradi et al., 2011). Nonetheless, it is crucial to note that these linkages exhibit non-linear characteristics and manifest inconsistency across distinct markers. Moreover, factors that introduce perturbations to ovarian functionality, encompassing instances such as ovarian surgery, autoimmune variations, smoking habits, and the utilization of androgenic exogenous hormones, have the potential to reverberate across the ovarian reserve. This resonance is characterized by diminished follicle counts and compromised oocyte quality and quantity. Consequently, this is closely aligned with an earlier onset of menopause and the initiation of ovarian senescence (Seyhan et al., 2015; Romanski et al., 2019).

Consequently, the computation of senescence-related genes (DEGs) is intricately linked with EM. This was accomplished through a co-expression network analysis of genes differentially expressed within the context of EM. Subsequently, leveraging the prowess of LASSO, SVM-RFE, and RF identified three candidate attributes: FLT1, LMNA, and BAK1. These attributes revealed insights from the gene selection process. Within this framework, the neural network model assumed a defining role, identifying the predictive weights attributed to the associated genes. This led to the establishment of a classification model score for EM. Finally, the correlation between the modeled genes and the immune characteristics of EM patients was explored.

Angiogenesis emerges as a plausible player in the intricate pathogenic fabric of EM, wherein FLT1—a prominent receptor for vascular endothelial growth factor (VEGF)—is pivotal as a major regulator of pro-angiogenic factors. Differential expression of FLT1 characterizes all subtypes of EM in comparison to their normal tissue counterparts. Furthermore, the emergence of high-risk red lesions may be attributed to elevated levels of VEGF and its receptors (FLT1 and FLT2), instigating an augmentation in the subperitoneal vascular network. This then fosters the propagation of malignant retroperitoneal gap implantation (Donnez et al., 1998; Aydin et al., 2021).

Changes in the hypermethylation status of the CpG islets of the laminin A/C (LMNA) gene are associated with insulin resistance in patients with polycystic ovary syndrome (PCOS), suggesting that this gene may be involved in the regulation of PCOS-associated insulin resistance (Ting et al., 2013). In addition, insulin resistance is an important pathological mechanism in endometrial cancer and EM (Wei and Li, 2020), and mutations in LMNA are thought to be a causal factor in non-valvular atrial fibrillation (NVAF) (Saj et al., 2012) and Hutchinson–Gilford premature senescence syndrome (HGPS) (Auld et al., 2010).

The Bcl-2 family gene BCL2 Antagonist/Killer 1 (BAK1) was found expressed at high levels in the apoptotic tissues of several clinical diseases (Zhou et al., 2008; Xu et al., 2011; Magee et al., 2014), and EM has been shown by some scholars to be a pathological consequence of inflammation and apoptosis (Laganà et al., 2019); thus, the high expression of BAK1 is involved in the pathomechanical process that regulates inflammation and apoptosis in the endometrium of EM patients.

The peritoneal immune milieu plays a pivotal role in the intricate EM pathophysiology (Vallvé-Juanico et al., 2019). This environment encompasses compromised natural killer cells, perturbed T-cell differentiation, and autoantibodies stemming from activated B cells (Chi et al., 2022b; Zhao et al., 2022; Chi et al., 2023c; Xiong et al., 2023). Consequently, it is critical to scrutinize the interplay that links senescence-associated prognostic genes with the diverse immune attributes exhibited by EM patients. This exploration is driven by the aim of formulating potential immunotherapeutic regimens or pharmaceutical agents for clinical implementation. Moreover, it seeks to enhance prevailing clinical treatment protocols, thereby heightening their overall efficacy and engendering superior therapeutic outcomes.

This investigation seeks to leverage machine learning algorithms to sift through the genetic signatures of EM to create a novel diagnostic framework grounded in artificial neural networks. Its ultimate objective is tangible benefits for clinical patients. However, it is important to acknowledge certain inherent limitations within this study. First is the relatively modest sample size within the cohort, which may not well capture the broader population dynamics, consequently influencing the applicability and generalization of the diagnostic model. Additionally, it is important to recognize that the diagnostic model is predicated on preliminary findings and lacks robust experimental validation essential to substantiate its clinical viability and dependability. Given these constraints, the diagnostic model necessitates further in-depth scrutiny and exploration to ascertain its potential utility in shaping clinical decision-making processes.

In conclusion, EM imposes an immeasurable burden beyond its localized symptoms, with far-reaching effects on all aspects of overall health. The chronic pain, reproductive challenges, and potential complications associated with it not only severely impact the physical health of patients but also their mental and emotional wellbeing, placing a heavy economic burden on society. We have identified a better diagnostic model than previously available (Ji et al., 2022), and these data highlight the need for further research to uncover senescence-related mechanisms in EM. It has laid some foundation and direction for more in-depth research afterward.

## 5 Conclusion

Within this investigation, a trio of pivotal senescence hub genes—BAK1, LMNA, and FLT1—were meticulously chosen. This selection was grounded in comprehensive bioinformatics analysis, and these hub genes were leveraged to create a “classifier” and forge a nomogram plot dedicated to endometriosis (EM). Furthermore, the study revealed the existence of two discernible senescence-associated subtypes, coupled with the discernment of pivotal regulatory pathways and the intricate landscape of the immune microenvironment. This research may deliver fresh perspectives concerning the future of drug interventions and the molecular underpinnings of EM.

## Data Availability

The datasets presented in this study can be found in online repositories. The names of the repository/repositories and accession number(s) can be found in the article/Supplementary Material.
