# Supplementary material for: Revealing the diagnostic value and immune infiltration of senescence-related genes in endometriosis: a combined single-cell and machine learning analysis
Source: Front Pharmacol. 2023 Oct 3;14:1259467. doi: 10.3389/fphar.2023.1259467 (PMC10583561; doi:10.3389/fphar.2023.1259467)
Supplement: Supplementary file 3 [file DataSheet1.DOCX]

Dear Editors,

We have resubmitted our manuscript and checked and uploaded all the raw data, codes, and images to the Jianguoyun. We hope you can easily find the source data through this link: https://www.jianguoyun.com/p/Dd1H8-cQqOfdCxjDso8FIAA.

If you have any questions, please feel free to contact me!

Have a nice day!

Best regards,

Lian Zou
